# A Rare Case of Chronic Uterine Inversion Secondary to Submucosal Fibroid Managed in the Province Hospital of Nepal

**DOI:** 10.1155/2020/6837961

**Published:** 2020-03-16

**Authors:** Amit Singh, Rajan Ghimire

**Affiliations:** ^1^Province Hospital, Karnali Province, Surkhet, Nepal; ^2^District Hospital, Terhathum, Nepal

## Abstract

Uterine inversion secondary to uterine pathology is a rare scenario that a gynecologist encounters. Unlike puerperal uterine inversion, it is misleading and may not always be possible to reduce to normal position without surgery. We report a case of a 35-year female with per vaginal bleeding for fifteen months with a mass-like sensation in the vaginal canal. She presented in shock and had a globular mass in the vaginal canal with the indistinct cervical os. She was resuscitated with blood transfusions and intravenous fluid. She was posted for emergency surgery where myomectomy was done vaginally, and finally, Haultain's procedure was carried out. The uterus was preserved.

## 1. Background

Uterine inversion is turning inside out of the fundus into the uterine cavity [1]. The commonest cause for uterine inversion is mismanagement of the third stage of labor . Nonpuerperal inversion of the uterus is rare, representing only one-sixth of all inversion cases [2]. The prolapsed uterine fibroid is a common nonpuerperal cause for uterine inversion, other causes being leiomyosarcoma, rhabdomyosarcoma, endometrial polyps, endometrial carcinoma, and uterovaginal prolapse [3].

Three contributing factors proposed for uterine inversion are (1) sudden emptying of the uterus, which was previously distended by a tumor, (2) thinning of the uterine walls due to an intrauterine tumor, and (3) dilatation of the cervix [4, 5]. We report a case of chronic uterine fibroid presenting with per vaginal bleeding for a long time. As there are only a handful of case reports available, we hope this case report will add up the scientific validity of the management procedure that is being carried out across the world. Also, this case was managed in a resource-poor setting without adequate imaging facilities like an MRI and limited blood products (whole blood only available) which certainly has challenges during the management of the case that we want to share here.

## 2. Case Report

In our case, a 35-year married female presented to emergency services with the complaint of bleeding per vagina and the feeling of something inside the vagina for 15 months. She used to have per vaginal bleeding with clots in one- to two-week interval which lasts for two to five days (no separate recognizable menstrual cycle). In between intermenstrual bleeding, she had a watery discharge from the vagina without any foul smell or per vaginal itch. Regarding per vaginal mass, she used to feel mass occasionally which stays inside the vaginal canal, never came out of introitus. There were postcoital bleeding and generalized weakness with no shortness of breath, palpitation, gum bleed, or rashes. She underwent minilaparotomy for permanent sterilization eleven years back and had recanalization done five years back. She delivered three children vaginally which were all home deliveries assisted by neighbors; last childbirth was twelve years back. During these fifteen months, she visited different health institutes where she was investigated for reasons of bleeding including coagulation profile, ultrasonography of the pelvis (all were normal), and prescribed progesterone, tranexamic acid, mefenamic acid, and treatment for vaginal discharge syndrome which could not help much to improve her problems.

On examination, she was well built, pale with vital signs within normal limits. The abdomen was soft and nontender. On bimanual per vaginal examination, approximately 6 × 4 cm firm globular mass was felt within the vaginal canal, separate cervical os could not be appreciated, the uterus could not be palpated, and there was active per vaginal bleeding.

**Figure 1:**
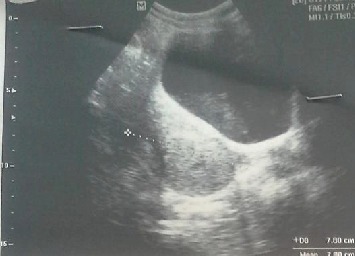
Ultrasonography of uterine inversion.

On investigation, her hemoglobin was 5.3 gm% and platelets and PT/INR were within the normal range. Ultrasonography (Figure 1) done by the radiologist in another center and at our center was reported normal. MRI could not be done as it was not available at our center and the patient needs to travel five to six hours for this, with so much of continuing per vaginal bleeding. Also, she did not afford the cost of an MRI. So, we decided to go for surgery without an MRI.

**Figure 2:**
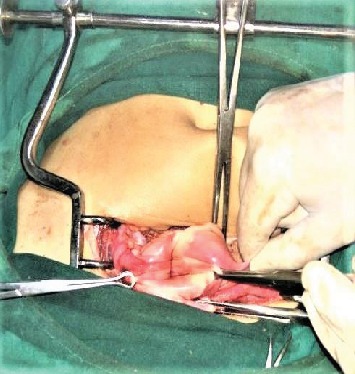
Cupping of the uterine content including fundus of the uterus, broad ligament, both ovaries, and round ligament.

**Figure 3:**
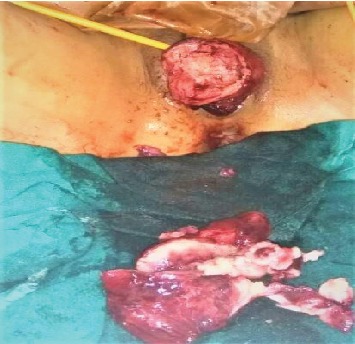
Picture after completion of vaginal myomectomy of the inverted uterus in piecemeal and before repair of myometrium and endometrium.

**Figure 4:**
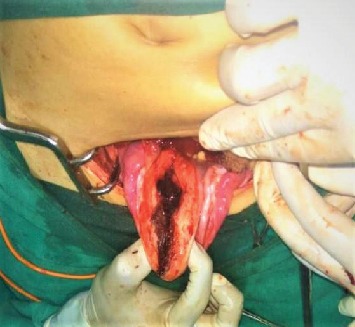
Haultain's procedure.

**Figure 5:**
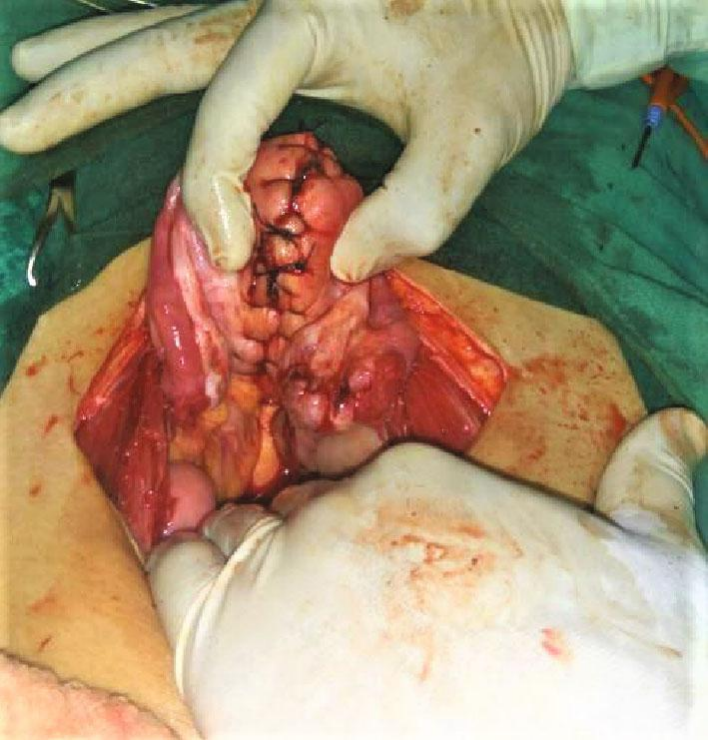
Final view of uterus repair.

She was resuscitated with intravenous fluid, and two units of whole blood as packed red blood cell were not available at the center. Ultrasonography was repeated in the preoperative room which was again unable to differentiate whether it was fibroid polyp or uterine inversion. She was planned for emergency operation with a provisional diagnosis of chronic inversion of uterus secondary to submucosal fibroid uterus (arising from fundus) with differential diagnosis of fibroid polyp. Haultain's procedure was planned. Foley's catheterization was done. With all standard precautions, the abdomen was opened in layers. A cup-like depression was noted in the midpelvic cavity, and bilateral round ligaments, fallopian tubes, and ovarian ligaments were coming out of the cup-like depression (Figure 2). A cyst of 5 × 6 cm in the right ovary was noted which got ruptured during the procedure, releasing serous fluid. Attempt to reduce uterine inversion abdominally was not successful. So, with the index finger of the assistant surgeon in the cup-like depression from the abdomen, the patient was repositioned in the lithotomy position; the mass was pulled out vaginally. With an index finger still in depression placed up to the fundus of the uterus from the abdominal cavity, myomectomy was done vaginally (Figure 3). The excised mass showed a whorled pattern consistent with uterine fibroid. Myometrium and endometrium were repaired. Again in the supine position of the patient, anterior and posterior edges of depression were held with Allis forceps. With sustained traction on the bilateral round ligament, a vertical incision was given in the posterior portion of depression (i.e., posterior uterus), fundus was pushed vaginally, and the uterine inversion was corrected (Figure 4). The posterior opening of the uterus was repaired (Figure 5). The ruptured ovarian cyst was removed. Approximately 30 percent of the ovary was preserved on the right side, and the left ovary was normal in appearance. The abdomen was closed in layers. The vaginal pack was kept for 24 hours. The specimen was not sent for histopathological examination as histopathological service was not available at our center and the patient could not afford to take the specimen to other centers. Besides, the gross examination of tissue was consistent with fibroid tissue.

The postoperative period was uneventful. One unit of whole blood was transfused in the postoperative ward. After total transfusion of three units of whole blood (two units preoperative and one-unit postoperative), her hemoglobin level on the third postoperative day was 7.4 gm%. She was counseled regarding the decreased chance of conception as she had undergone tubal recanalization for tubal ligation done in the past. Also, she had an ovarian cyst which was removed this time with ovarian reserve of 30 percent on the right side which also decreases the chance of conception. We advised not to conceive for at least two to three years, and if she got pregnant, she will need to undergo an elective cesarean section. She was discharged on the seventh postoperative period with iron tablets.

## 3. Discussion

Uterine inversion is a rare event either puerperal or nonpuerperal, with only few case reports. This may be because the cases are missed if the inversion is not complete and is chronic. The same thing happened with our case too. She visited multiple primary and tertiary level centers with the complaint of per vaginal bleeding for fifteen months. She visited our center in hypovolemic shock secondary to per vaginal bleeding. She had stage 2 uterine inversion (complete inversion of the uterine fundus through the fibromuscular cervix) [6].

The degree of inversion can be classified into incomplete, complete, or total. In the incomplete form, the uterine fundus descends inferiorly but not through the cervix. In a complete inversion, fundus and corpus extend through the cervix. In a total inversion, the vagina is also inverted [1, 7]. In our case, it was a complete type. Diagnosis is always not easy. Acute forms are mostly symptomatic while chronic form can be asymptomatic or associated with pelvic pain with a sensation of heaviness or per vaginal bleeding. On physical examination, a vaginal mass can be detected, but the uterus is not palpable by bimanual examination [7].

The ultrasonographic examination should include sagittal images across the entire uterus with proper cephalocaudal orientation since images that are not oriented correctly may be misleading and could give the false impression of the normal anatomic position of the uterus. The ultrasonographic finding includes an indentation of the cephalad portion of the uterus with a hypoechoic structure (ovary) on top that seemed to be protruding from the indentation [8]. In our case, multiple attempts of ultrasound were done including in the operation theatre until we could conclude for uterine inversion.

The manual reduction can be tried with varying degrees of success rate. The hydrostatic method of reduction had also been tried in a few cases of acute inversion [1]. We decided on Haultain's operation in our patient where the cervical ring is incised posteriorly with a longitudinal incision [9]. With the help of two Allis forceps applied onto the crater on each side, gentle upward traction exerted on the forceps. Though not always required, the upward thrust was given vaginally to the inverted fundus of uterus till the uterus is restored to the normal anatomic position. Another way of correcting the uterine inversion is the Huntington procedure where the abdomen is opened, and the inversion site is exposed. Applying two Allis forceps over each side of the crater, gentle upward traction is applied and with other forceps placed on advancing fundus; the uterus is restored to normal anatomic position [10]. The Kustner and Spinelli vaginal approach procedures could also be used. The Kustner procedure is to enter the pouch of Douglas vaginally and to split the posterior aspect of the uterus and the cervix for reinverting the uterus. In Spinelli operation, an incision is made on the anterior aspect of the cervix and then the uterus is reinverted [11]. Many a time, reposition of the uterus is not possible, and the hysterectomy has to be done. In our case, we could save the uterus, fallopian tubes, and ovaries.

All cases of uterine inversion are not always straight forward. Sometimes it is challenging to diagnose the case timely. When the case is diagnosed also, there can be a challenge to manage and has to be operated with multiple approaches like abdominal and vaginal approach. This case taught us to think of uterine inversion in a patient with per vaginal bleeding and mass-like feeling in the vaginal canal even though ultrasonographic findings are normal.

## 4. Conclusion

Uterine inversion secondary to uterine pathology is a very rare condition because of which it is easily missed. If timely managed, it has an excellent prognosis. Though nonsurgical approaches can be tried, surgical procedures are ultimate answers.

## References

[1] Bhalla R., Wuntakal R., Odejinmi F., Khan R. U. (2009). Review acute inversion of the uterus. *Obstetrics and Gynecology*.

[2] Takano K., Ichikawa Y., Tsunoda H., Nishida M. (2001). Uterine inversion caused by uterine sarcoma : a case report. *Japanese Journal of Clinical Oncology*.

[3] Katdare P., Valecha S. M., Gandhewar M., Dhingra D. (2013). Chronic non - peurperal uterine inversion: recommendations for diagnosis and management. *Global Journal of Medical Research*.

[4] Lascarides E., Cohen M. (1968). Surgical management of nonpuerperal inversion of the uterus. *Obstetrics & Gynecology*.

[5] Kumari A., Vidhyarthi A., Salini K. M. (2016). Chronic uterine inversion secondary to submucous fibroid: a rare case report. *International Journal of Scientific Study*.

[6] Skinner G. N., Louden K. A. (2001). Non-puerperal uterine inversion associated with an atypical leiomyoma. *The Australian and New Zealand Journal of Obstetrics and Gynaecology*.

[7] Leconte I., Thierry C., Bongiorno A., Luyckx M., Fellah L. (2016). Non-puerperal uterine inversion. *Journal of the Belgian Society of Radiology*.

[8] Smulian J. C., DeFulvio J. D., Diven L., Terrazas J. L. (2013). Sonographic findings in acute uterine inversion. *Journal of Clinical Ultrasound*.

[9] Haultain F. W. N. (1901). The treatment of chronic uterine inversion by abdominal hysterotomy, with a successful case. *The British Medical Journal*.

[10] Huntington J. L., Irving F. C., Kellogg F. S. (1928). Abdominal reposition in acute inversion of the puerperal uterus. *American Journal of Obstetrics and Gynecology*.

[11] Birge O., Tekin B., Merdin A., Coban O., Arslan D. (2015). Chronic total uterine inversion in a young adult patient. *American Journal of Case Reports*.

